# Supplementary material for: Resolving the full spectrum of human genome variation using Linked-Reads
Source: Genome Res. 2019 Apr;29(4):635–45. doi: 10.1101/gr.234443.118 (PMC6442396; doi:10.1101/gr.234443.118)
Supplement: Supplemental Material [file supp_gr.234443.118_Supplemental_longranger-2.2.zip › longranger-2.2.tar.bz2!/lr2.2-public/lib/stan/cmdstan/stan/doc/api/html/precomputed__gradients_8hpp_source.html]

Stan: src/stan/agrad/rev/internal/precomputed\_gradients.hpp Source File


|  |  |
| --- | --- |
|  | Stan  2.5.0  probability, sampling & optimization |


- Main Page
- Related Pages
- Namespaces
- Classes
- Files

- File List
- File Members

All Classes Namespaces Files Functions Variables Typedefs Enumerations Enumerator Friends Macros Pages

- src
- stan
- agrad
- rev
- internal

precomputed\_gradients.hpp

Go to the documentation of this file.

1 #ifndef STAN\_\_AGRAD\_\_REV\_\_INTERNAL\_\_PRECOMPUTED\_GRADIENTS\_HPP

2 #define STAN\_\_AGRAD\_\_REV\_\_INTERNAL\_\_PRECOMPUTED\_GRADIENTS\_HPP

3

4 #include <iostream>

5 #include <vector>

6 #include <stdexcept>

7 #include <stan/agrad/rev/vari.hpp>

8 #include <stan/agrad/rev/var.hpp>

9

10 namespace stan {

11  namespace agrad {

12

20  class precomputed\_gradients\_vari : public vari {

21  protected:

22  std::vector<vari \*> varis\_;

23  std::vector<double> gradients\_;

24

25  public:

41  precomputed\_gradients\_vari(const double val,

42  std::vector<vari \*>& varis,

43  const std::vector<double>& gradients)

44  : vari(val),

45  varis\_(varis),

46  gradients\_(gradients) {

47  if (varis\_.size() != gradients\_.size())

48  throw std::invalid\_argument("sizes of varis and gradients do not match");

49  }

50

58  void chain() {

59  for (size\_t n = 0; n < varis\_.size(); n++) {

60  varis\_[n]->adj\_ += adj\_ \* gradients\_[n];

61  }

62  }

63  };

64

65

86  var precomputed\_gradients(const double value,

87  const std::vector<var>& vars,

88  const std::vector<double>& gradients) {

89  std::vector<vari \*> varis;

90  varis.resize(vars.size());

91  for (size\_t n = 0; n < vars.size(); n++) {

92  varis[n] = vars[n].vi\_;

93  }

94  return var(new precomputed\_gradients\_vari(value, varis, gradients));

95  }

96  }

97 }

98 #endif

stan::agrad::precomputed\_gradients\_vari

This is a var implementation class that takes precomputed gradient values.

**Definition:** precomputed\_gradients.hpp:20

vari.hpp

stan::agrad::precomputed\_gradients\_vari::gradients\_

std::vector< double > gradients\_

**Definition:** precomputed\_gradients.hpp:23

stan::agrad::precomputed\_gradients

var precomputed\_gradients(const double value, const std::vector< var > &vars, const std::vector< double > &gradients)

This function is provided for Stan users that want to compute gradients without using Stan's auto-dif...

**Definition:** precomputed\_gradients.hpp:86

stan::agrad::precomputed\_gradients\_vari::chain

void chain()

Implements the chain rule for this variable.

**Definition:** precomputed\_gradients.hpp:58

stan::agrad::var::vi\_

vari \* vi\_

Pointer to the implementation of this variable.

**Definition:** var.hpp:40

stan::agrad::precomputed\_gradients\_vari::varis\_

std::vector< vari \* > varis\_

**Definition:** precomputed\_gradients.hpp:22

stan::agrad::vari

The variable implementation base class.

**Definition:** vari.hpp:28

var.hpp

stan::agrad::var

Independent (input) and dependent (output) variables for gradients.

**Definition:** var.hpp:27

stan::agrad::precomputed\_gradients\_vari::precomputed\_gradients\_vari

precomputed\_gradients\_vari(const double val, std::vector< vari \* > &varis, const std::vector< double > &gradients)

Constructs a precomputed\_gradients\_vari.

**Definition:** precomputed\_gradients.hpp:41

stan::agrad::vari::adj\_

double adj\_

The adjoint of this variable, which is the partial derivative of this variable with respect to the ro...

**Definition:** vari.hpp:43


---

[ Stan Home Page ]

*© 2011–2014,
Stan Development Team.*
